# Supplementary material for: Empirical methods for controlling false positives and estimating confidence in ChIP-Seq peaks
Source: BMC Bioinformatics. 2008 Dec 5;9:523. doi: 10.1186/1471-2105-9-523 (PMC2628906; doi:10.1186/1471-2105-9-523)
Supplement: Additional File 1 — A variety of html documents from the USeq web site detailing the available applications, their best usage, output file type descriptions, command line menus, etc. [file 1471-2105-9-523-S1.zip › USeqUserGuides/JavaDoc.zip!/JavaDoc/util/gen/SpearmanCorrelation.html]

SpearmanCorrelation


|  |  |  |
| --- | --- | --- |
| **Overview** \| **Package** \| **Class** \| **Use** \| **Tree** \| **Deprecated** \| **Index** \| **Help** | |  |
| **PREV CLASS**   **NEXT CLASS** | **FRAMES**    **NO FRAMES**     **All Classes** |  |
| SUMMARY: NESTED \| FIELD \| CONSTR \| METHOD | DETAIL: FIELD \| CONSTR \| METHOD |  |


---


## util.gen Class SpearmanCorrelation

```
java.lang.Object
  util.gen.SpearmanCorrelation
```

---

``` public class SpearmanCorrelation extends java.lang.Object ```

Calculates a Spearman correlation coefficient (rho), in the case of ties, averages the ranks
and returns a Pearson correlation coefficient on the ranks.

**Author:**
:   Nix

---

**Constructor Summary**

`SpearmanCorrelation()`


| **Method Summary** | |
| --- | --- |
| `double` | `corrCoeff(float[] a, float[] b)`             This is the final step in calculating a Spearman Correlation coeff. |
| `RankedFloatArray` | `getARFA()` |
| `RankedFloatArray` | `getBRFA()` |
| `static void` | `main(java.lang.String[] args)` |
| `RankedFloatArray` | `rank(float[] f)` |
| `RankedFloatArray` | `rankSamples(RankSample[] rs)`             Ranks a sorted array of RankSample based on value. |
| `void` | `setARFA(RankedFloatArray arfa)` |
| `void` | `setBRFA(RankedFloatArray brfa)` |
| `double` | `spearmanCorrelationCoefficient(float[] a, float[] b)`             Returns a Spearman Rank Correlation Coefficient (rho) between the two arrays. |
| `double` | `spearmanCorrelationCoefficient(RankedFloatArray aRFA, RankedFloatArray bRFA)`             Returns a Spearman Rank Correlation Coefficient (rho) between the two arrays. |

**Methods inherited from class java.lang.Object**

`equals, getClass, hashCode, notify, notifyAll, toString, wait, wait, wait`

**Constructor Detail**

### SpearmanCorrelation

```
public SpearmanCorrelation()
```


**Method Detail**

### spearmanCorrelationCoefficient

```
public double spearmanCorrelationCoefficient(float[] a,
                                             float[] b)
```

:   Returns a Spearman Rank Correlation Coefficient (rho) between the
    two arrays. If ties are found, their ranks are averaged and a
    Pearson correlation is calculated on the ranks.

---


### spearmanCorrelationCoefficient

```
public double spearmanCorrelationCoefficient(RankedFloatArray aRFA,
                                             RankedFloatArray bRFA)
```

:   Returns a Spearman Rank Correlation Coefficient (rho) between the
    two arrays. If ties are found, their ranks are averaged and a
    Pearson correlation is calculated on the ranks.

---


### corrCoeff

```
public double corrCoeff(float[] a,
                        float[] b)
```

:   This is the final step in calculating a Spearman Correlation coeff.
    Don't use it unless you know what you are doing

    :   **Parameters:**: `a` - - a values converted to ranked and sorted by original index: `b` - - ditto Call correlate() if you don't have the ranks.

---


### rank

```
public RankedFloatArray rank(float[] f)
```

---


### rankSamples

```
public RankedFloatArray rankSamples(RankSample[] rs)
```

:   Ranks a sorted array of RankSample based on value.
    If no ties are found then this is simply their array index number+1.
    (ie 1,2,3,4...)
    If ties are encountered, ties are assigned the average of their index
    positions+1. (ie if index+1's: 2,3,4 have the same absolute difference, all are assigned a
    rank of 3).

---


### main

```
public static void main(java.lang.String[] args)
```

---


### getARFA

```
public RankedFloatArray getARFA()
```

---


### setARFA

```
public void setARFA(RankedFloatArray arfa)
```

---


### getBRFA

```
public RankedFloatArray getBRFA()
```

---


### setBRFA

```
public void setBRFA(RankedFloatArray brfa)
```


---


|  |  |  |
| --- | --- | --- |
| **Overview** \| **Package** \| **Class** \| **Use** \| **Tree** \| **Deprecated** \| **Index** \| **Help** | |  |
| **PREV CLASS**   **NEXT CLASS** | **FRAMES**    **NO FRAMES**     **All Classes** |  |
| SUMMARY: NESTED \| FIELD \| CONSTR \| METHOD | DETAIL: FIELD \| CONSTR \| METHOD |  |


---
